# Thresholds for vestibular and cutaneous perception and oculomotor response induced by galvanic vestibular stimulation

**DOI:** 10.3389/fneur.2022.955088

**Published:** 2022-08-12

**Authors:** Thanh Tin Nguyen, Jin-Ju Kang, Sun-Young Oh

**Affiliations:** ^1^Jeonbuk National University College of Medicine, Jeonju, South Korea; ^2^Department of Neurology, Jeonbuk National University Hospital and School of Medicine, Jeonju, South Korea; ^3^Department of Pharmacology, Hue University of Medicine and Pharmacy, Hue University, Hue, Vietnam; ^4^Research Institute of Clinical Medicine of Jeonbuk National University-Biomedical Research Institute of Jeonbuk National University Hospital, Jeonju, South Korea

**Keywords:** galvanic vestibular stimulation (GVS), vestibular perception threshold, cutaneous threshold, nystagmus, vertigo

## Abstract

**Objectives:**

In this study, the specific threshold intensities and response characteristics of galvanic vestibular stimulation (GVS) on vestibular (conscious) and cutaneous (detrimental) perception as well as oculomotor nystagmus (reflex) were determined.

**Methods:**

The threshold intensities for vestibular and cutaneous perception and oculomotor response induced by GVS were determined in 25 right-handed healthy subjects (32.6 ± 7.2 years of age; 56% female). The subjects were seated upright, and eye movements were recorded while a direct GVS current was applied with paradigms of cathode on the right and anode on the left (CRAL) and also cathode on the left and anode on the right (CLAR).

**Results:**

Subjects experienced dizziness, sense of spinning, or fall tendency, which was more frequently directed to the cathode (76%) than the anode (24%, *p* < 0.001, chi-square one-variable test) at mean current greater than 0.98 ± 0.29 mA (mean vestibular threshold). The current also triggered a more frequent mild tingling sensation at the cathode (56%) than the anode (30%) or on both sides (14%; *p* = 0.001, chi-square one-variable test) when above the mean cutaneous threshold of 0.9 ± 0.29 mA. Above the mean oculomotor threshold of 1.61 ± 0.35 mA, combined horizontal and torsional nystagmus was more frequent toward the cathode (86%) than toward the anode (*p* < 0.001, chi-square one-variable test). The mean oculomotor threshold was significantly higher than both the vestibular (*p* < 0.001, Mann–Whitney *U*-test) and cutaneous (*p* < 0.001, Mann–Whitney *U*-test) thresholds, which were comparable (*p* = 0.317, Mann–Whitney *U*-test). There was no significant disparity in these specific thresholds between the two GVS paradigms. The vestibular threshold was significantly higher in males than in females [1 (0.5–1.25) mA vs. 0.75 (0.625–1.125) mA, *Z* = −2.241, *p* = 0.025, Mann–Whitney *U*-test]. However, the thresholds of cutaneous perception and oculomotor response did not differ by sex.

**Conclusion:**

The findings indicate that thresholds for vestibular and somatosensory perception are lower than the oculomotor threshold. Therefore, a strategy to reduce GVS current intensity to the level of vestibular or somatosensory perception threshold could elicit beneficial vestibular effects while avoiding undesirable effects such as oculomotor consequences.

## Introduction

Vestibular afferents are sensitive to motion acceleration when the head translates and rotates in space, creating rapid and accurate reflexive responses to unpredictable and high acceleration. This function is critical in maintaining balance and visual stability (1, 2). The afferents provide continuous data for exploring and comprehending a vast range of physical motions encountered in daily life, acting as an inertial sensor and significantly contributing to spatial navigation (1). Notably, the vestibular system is multimodal and integrative (1, 3, 4), involving the fusion of otolith- and canal-derived signals at the level of vestibular nuclei and multisensory integration and convergence (such as vestibular-visual, vestibular-somatosensory, and vestibular-visual-somatosensory interactions) at the vestibular nuclei, thalamus, and cerebral cortices (1, 3, 4). The coherence integration of three systems (i.e., vestibular, visual, and somatosensory) imparts the ability to determine an internal representation of space and spatial perception and navigation in three-dimensional coordinates, using both egocentric and exocentric strategies (5, 6). The interaction between body and environmental space *via* navigation continuously occurs based on data from the navigable space (optic flow and visual cues) and the perception of self-motion (vestibular and somatosensory) (5, 6).

Over the past century, transcutaneous delivery of electric currents to the vestibular afferents, commonly referred to as galvanic vestibular stimulation (GVS), has been used to study and understand the function of the vestibular system (7). GVS is a technique that can stimulate the spike trigger zone of primary vestibular afferents associated with both semicircular canals (SCCs) and otolith organs (8, 9), allowing perception of vestibular sensations with excellent temporal control (10, 11). Firing rates of peripheral vestibular afferents are increased by galvanic currents at the cathode and decreased at the anode (9, 12, 13). The adaptive capacity of two types of vestibular afferent fibers to each GVS waveform is strikingly different that irregular fibers are sensitive to small-amplitude, highly dynamic currents (alternating current, AC), but fail to sustain tonic firing rates with constant currents (direct current, DC), whereas regular fibers are the opposite (14). GVS has also been used in functional neuroimaging studies to investigate the vestibular system (15) and in a variety of behavioral experiments on the effects of vestibular stimulation on locomotion and cognitive processes (16). In the framework of clinical therapeutic studies, favorable advantages of GVS have been shown in the improvement of postural stability (17–19), gait performance (20), functional mobility (21, 22), sensory perception (23–25), and cognition (26, 27).

Due to an increasing interest in GVS applications in clinical neuropsychiatry and rehabilitation, it is important to determine the stimulus parameters of waveforms (DC, sinusoidal or noisy), intensity, frequency, polarity, duration, and timing of stimulation, as well as electrode size and positioning (14). When altering these parameters, different amounts of electric current can be elicited, and diverse physiologic and adverse effects induced (14). In addition to the assessment of efficacy, electrical safety is crucial for the clinical application of GVS and must be considered in the intervention. Frequently reported detrimental effects of GVS include itching, tingling, and burning at the stimulation site. In addition, high GVS amplitude leads to significantly decreased performance in short-term spatial memory or egocentric mental rotation, supported by a significant decrease in cell proliferation in the hippocampus (28), induced postural instability (29), and decreased dynamic visual acuity (30). To achieve the optimal effect and simultaneously reduce detrimental effects, it is necessary to determine the GVS threshold defined by the lowest level of stimulus required to evoke vestibular responses. GVS currents also activate other sensory inputs including those of the cutaneous, visual, and auditory systems as well as the vestibular system, which encompasses somatosensory, oculomotor, and vestibular responses during an intervention (14). Therefore, investigating the thresholds for specific points of each sensory perspective, cutaneous and vestibular perception as well as oculomotor response, is necessary. Because precise sensory feedback from the visual, vestibular, and cutaneous systems is critical for maintaining balance and motor control, determining the appropriate individualized GVS threshold will maximize the therapeutic effects of GVS intervention while minimizing the associated unpleasant sensations. In particular, due to increased clinical application of GVS, the threshold should be determined prior to the experimental session (31–33). Previous research has determined GVS threshold based on mostly vestibular perception, occasionally cutaneous feeling, and rarely oculomotor activity. To the best of our knowledge, no study has previously assessed these specific GVS thresholds concurrently. Thus, the most appropriate method to determine GVS threshold remains unclear. In this study, specific threshold intensities and response characteristics of GVS on vestibular and cutaneous perception as well as oculomotor response were determined.

## Materials and methods

### Participants

This study included 25 healthy subjects (32.6 ± 7.2 years; range 23–53 years; 11 males, 14 females, Table 1) recruited at Jeonbuk National University Hospital from July to September 2021. According to the Edinburgh Handedness Inventory (34), all participants were right-handed. Participants did not have any history of neurologic or neuro-otologic diseases and were not taking any medication. The subjects underwent neurotological evaluations including video oculography and head-impulse test and vestibular-evoked myogenic potential tests to screen for any vestibular impairment. They showed no abnormalities on examinations. The subjects provided written informed consent to participate in the study following an explanation of the study protocol. All experiments were reviewed and approved by the Institutional Review Board at Jeonbuk National University Hospital (No. 2021-07-013-007).

**Table 1:** Thresholds for somatosensory and vestibular perception and oculomotor response and their characteristics in response to GVS with the cathode on the right and the anode on the left (CRAL).

**Pts./sex/age**	**Handedness**	**Cutaneous perception**		**Vestibular perception**		**Oculomotor response**			
		**Threshold (mA)**	**Sensing site**	**Threshold (mA)**	**Description**	**Threshold (mA)**	**Degree (°/s)**	**Duration (s)**	**Direction**
1/F/48	Rt.	0.5	Cathode (Rt.)	0.5	Rt. tilting and CW rotation, vertigo	1	2.1	5	RBN
2/F/36	Rt.	1	Cathode (Rt.)	1	Rt. tilting and CW rotation, vertigo	0.5	2.1	4.9	LBN
3/M/34	Rt.	0.5	Both	1	Rt. tilting and CW rotation,	1	2.5	5.4	RBN
4/M/33	Rt.	1	Cathode (Rt.)	1.5	Rt. tilting and CW rotation, vertigo	1.5	2	4.1	RBN
5/F/32	Rt.	0.5	Cathode (Rt.)	0.5	Rt. tilting and CW rotation, vertigo	2	1.8	4.3	RBN
6/M/34	Rt.	1	Cathode (Rt.)	2	CCW rotation	1.5	1.7	3.4	LBN
7/F/29	Rt.	1.5	Cathode (Rt.)	0.5	Rt. tilting and CW rotation	2	1.6	3.9	RBN
8/M/29	Rt.	1	Cathode (Rt.)	1	Lt. tilting and CCW rotation, vertigo	2	2.4	4.4	CW
9/M31	Rt.	0.5	Cathode (Rt.)	1	CW rotation	1	1.7	5.1	RBN
10/F/30	Rt.	1.5	Cathode (Rt.)	1	Lt. tilting and CCW rotation, vertigo	0.5	1.9	5	RBN
11/F/23	Rt.	1	Anode (Lt.)	0.5	Rt. tilting and CW rotation, vertigo	1.75	1.9	4.4	RBN
12/F/26	Rt.	1.5	Cathode (Rt.)	0.5	Rt. tilting and CW rotation, vertigo	1	2.5	5	CCW
13/F/25	Rt.	0.5	Both	0.5	CCW rotation	0.5	2.4	5.2	RBN
14/F/38	Rt.	0.5	Anode (Lt.)	0.5	Lt. tilting and CCW rotation, vertigo	1.5	1.8	4.5	CCW
15/F/44	Rt.	0.5	Both	0.5	Rt. tilting and CW rotation, vertigo	2	2.5	4.9	RBN
16/M/53	Rt.	1.25	Anode (Lt.)	1.5	Lt. tilting feeling	2	1.8	6	RBN
17/M/35	Rt.	0.5	Anode (Lt.)	1	CW rotation and vertigo	2	2.1	3.8	RBN
18/F/31	Rt.	0.5	Anode (Lt.)	0.75	Rt. tilting and CW rotation	1	2.3	8.7	RBN
19/F/26	Rt.	0.5	Cathode (Rt.)	0.75	Rt. tilting and CW rotation	2	1.7	5	LBN
20/F/28	Rt.	2	Cathode (Rt.)	2	Rt. tilting and CW rotation	2	1.6	4.2	RBN
21/M/25	Rt.	1.5	Cathode (Rt.)	1.75	CCW rotation	2	1.9	6.3	RBN
22/M/36	Rt.	1.25	Anode (Lt.)	1.25	Rt. tilting and CW rotation	2	2.2	5.8	RBN
23/M/33	Rt.	2	Cathode (Rt.)	2	Rt. tilting and vertigo	2	1.8	3.7	RBN
24/F/29	Rt.	1.25	Anode (Lt.)	1.25	Rt. tilting and CW rotation	1.75	1.7	4.3	RBN
25/M/27	Rt.	0.5	Anode (Lt.)	1	Rt. tilting and CW rotation	1.5	2.7	8	RBN, UBN

*GVS, galvanic vestibular stimulation; Pts, participants; M, male; F, female; Rt., right; Lt., left; CW, clockwise; CCW, counter-clockwise; RBN, right-beating nystagmus; LBN, left-beating nystagmus*.

### Experimental design and GVS procedure

**Figure 1:**
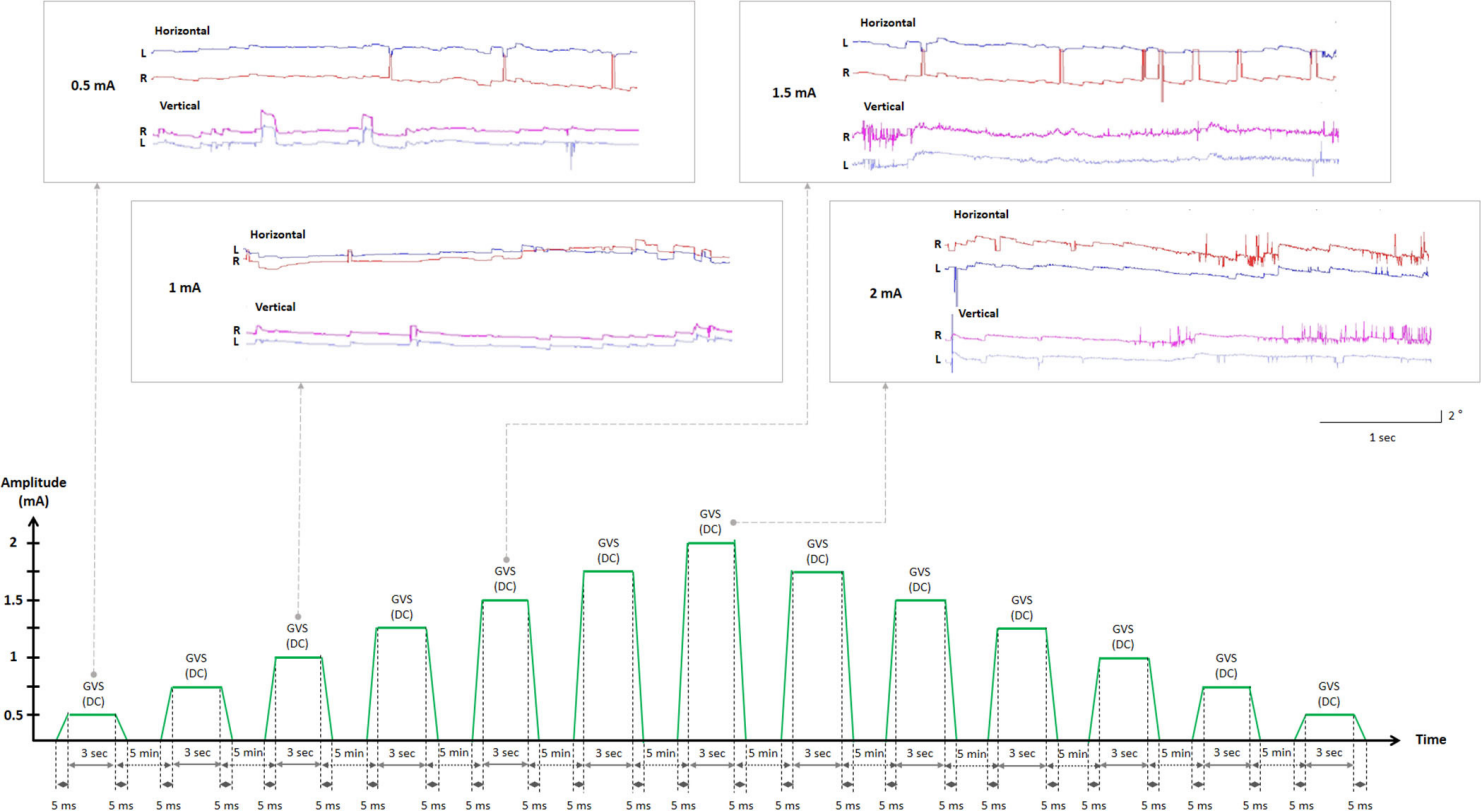
Experimental design flowchart. Representations of ocular movements during GVS application (in the boxes). The two-step approach to determining GVS thresholds. GVS, galvanic vestibular stimulation; DC, direct current; L, left eye (blue); R, right eye (red).

Data from each participant were collected in two randomly ordered sessions of DC-GVS paradigms of cathode on the right and anode on the left (CRAL) or cathode on the left and anode on the right (CLAR; Tables 1, 2). The experimental design using “CLAR and CRAL” in right-handed participants is required in the context of handedness-dependent vestibular lateralization, in which the vestibular input and signaling pathways from the dominant side are more predominant than those from the non-dominant side (35, 36). This allowed the detection of any difference in the GVS threshold between CLAR and CRAL models. At the beginning of the sessions, participants received verbal and written instructions regarding the tasks. A CE-certified battery-driven constant current stimulator (neuroConn DC-Stimulator Plus; neuroConn, Ilmenau, Germany) was used to deliver the DC to subjects sitting upright in a chair *via* a pair of 35 cm^2^ (5 × 7 cm) (37–39) rectangular conductive rubber electrodes (neuroConn) coated with electrode gel and placed binaurally over both mastoids. The maximum current density in this study was estimated to be 57.14 μA/cm^2^ (corresponding to a charge density of 1.71 Coloumb/cm^2^) at the skin surface, similar to previous studies (37, 38), and well below the safety limit for tissue damage (40–42). The electrodes were positioned on the mastoid, supplemented with a conductive gel prior to testing to reduce skin impedance, and secured in place by a rubber head strap. Participants were seated in a comfortable chair in front of a target bar of the three-dimensional video oculography instrument (3D-VOG, SMI, the Netherlands, sampling rate of 60 Hz) and wore goggles that tracked their ocular movements under the supervision of a senior neurotologist (S.Y. Oh). Threshold testing started with a low current (0.5 mA), gradually increasing by 0.25 mA for a 3-s period over a 5-min inter-step interval. In the inter-step interval, the subjects were asked to describe whether they experienced any tingling/pain (cutaneous perception threshold) or dizziness/unsteadiness (vestibular perception threshold) during GVS application. The senior neurotologist also monitored for the appearance of nystagmus when recording with the VOG (oculomotor threshold). These iterations were continued with a steady increase in current until the participant concurrently expressed tingling/pain, dizziness/unsteadiness, and nystagmus. To confirm the specific thresholds, the stimulus intensity was gradually decreased by 0.25 mA from 2 mA to the level at which the tingling/pain (cutaneous threshold), dizziness/unsteadiness (vestibular threshold), and ocular responses (oculomotor threshold) disappeared (Figure 1). The procedure was repeated two times to confirm the cutaneous, vestibular, and oculomotor thresholds (43–45). When thresholds differed between sessions, the mean value was used for analysis.

**Table 2:** Thresholds for somatosensory and vestibular perception and oculomotor response and their characteristics in response to GVS with the cathode on the left and the anode on the right (CLAR).

**Pts./sex/age**	**Handedness**	**Cutaneous perception**		**Vestibular perception**		**Oculomotor response**		
		**Threshold (mA)**	**Sensing site**	**Threshold (mA)**	**Description**	**Threshold (mA)**	**Degree (°/s)**	**Duration (s)**	**Direction**
1/F/48	Rt.	0.5	Cathode (Lt.)	0.5	Lt. tilting and CCW rotation	1.5	2.2	5.1	LBN
2/F/36	Rt.	0.5	Cathode (Lt.)	0.75	Lt. tilting and CCW rotation	1	2.7	5.7	LBN
3/M/34	Rt.	0.5	Cathode (Lt.)	0.5	Lt. tilting and CCW rotation, vertigo	1.5	1.5	4.9	LBN
4/M/33	Rt.	1	Anode (Rt.)	0.75	Lt. tilting and CCW rotation, vertigo	1.75	1.9	4.6	LBN
5/F/32	Rt.	1.25	Cathode (Lt.)	1.25	Lt. tilting and CCW rotation	2	1.8	3.8	LBN
6/M/34	Rt.	1.5	Both	1.25	Rt. tilting and CW rotation	1.5	2.1	4.5	RBN
7/F/29	Rt.	0.5	Cathode (Lt.)	0.5	Lt. tilting and CCW rotation	1.5	2.3	5	LBN
8/M/29	Rt.	0.75	Cathode (Lt.)	0.75	Lt. tilting and CCW rotation	1.75	2.4	4	LBN
9/M31	Rt.	1	Anode (Rt.)	1.25	Lt. tilting and CCW rotation	2	1.4	6	LBN
10/F/30	Rt.	1	Anode (Rt.)	1	Rt. tilting and CW rotation	1.5	1.8	4.4	LBN
11/F/23	Rt.	0.5	Cathode (Lt.)	1	Lt. tilting and CCW rotation	1.5	2.2	5	LBN
12/F/26	Rt.	1.25	Cathode (Lt.)	1.5	Rt. tilting and CW rotation, vertigo	2	2.6	4.8	LBN
13/F/25	Rt.	1	Cathode (Lt.)	1	Lt. tilting and CCW rotation	1.5	2	5.9	LBN
14/F/38	Rt.	0.75	Cathode (Lt.)	0.75	Lt. tilting and CCW rotation	2	2.7	6	LBN
15/F/44	Rt.	1	Both	1	Lt. tilting and CCW rotation, vertigo	2	2	4.5	LBN, DBN, CCW
16/M/53	Rt.	0.75	Cathode (Lt.)	0.75	CCW rotation	1.5	1.1	4.7	LBN
17/M/35	Rt.	0.5	Anode (Rt.)	1	Lt. tilting and vertigo	2	1.8	3	LBN, DBN
18/F/31	Rt.	0.5	Cathode (Lt.)	0.75	Lt. tilting and CCW rotation	1.5	2.3	6	LBN
19/F/26	Rt.	1	Anode (Rt.)	1.25	Rt. tilting and CW rotation	1.5	1.8	5	LBN
20/F/28	Rt.	0.5	Both	0.5	Lt. tilting and CCW rotation, vertigo	2	1.7	4	LBN
21/M/25	Rt.	1	Cathode (Lt.)	1	Lt. tilting and CCW rotation	1.5	1.2	6.1	LBN
22/M/36	Rt.	1.75	Cathode (Lt.)	1.75	Lt. tilting and CCW rotation	2	2	6.2	LBN
23/M/33	Rt.	0.5	Both	0.75	Lt. tilting and CCW rotation	2	2.6	5.3	LBN
24/F/29	Rt.	1	Anode (Rt.)	1.25	Lt. tilting and CCW rotation	1.5	2.1	7.7	RBN
25/M/27	Rt.	0.75	Anode (Rt.)	0.5	Rt. tilting and CW rotation	2	1.8	4.5	LBN

### Thresholds for vestibular and cutaneous perception and oculomotor response

A verbal warning preceded each stimulus, and subjects were instructed to say “yes” when perceiving tingling, pain, or any other sensation at the electrodes. Subjects were asked to describe the sensory feelings and indicate the location of occurrence. Subjects were also instructed to focus on vestibular perception and asked to report any sense of dizziness with spinning or non-spinning and disequilibrium or any sense of motion. Each report was followed by feedback confirming the perceptions such as motion direction, and the current intensity was increased in the next step. Eye position and movement were binocularly recorded using the 3D-VOG during the entire experiment. Digitized data were analyzed using the MATLAB software (MathWorks, Natick, MA, USA). All data pertaining to the results reported in this article (text and figures) will be shared.

### Statistical analysis

All data were analyzed using SPSS Statistics version 23.0 (IBM Corp., Armonk, NY, USA). For each parameter, the normality of the distribution was assessed using the Shapiro-Wilk test. The variables were presented as mean ± standard deviation. The non-parametric variables were indicated as median (95% confidence interval), and the significant difference was determined using the Kruskal-Wallis test (between group comparison) and the Mann-Whitney *U*-test (pairwise comparisons). The chi-square one-variable test was used to compare the difference between variables in multiple categories. The Spearman's correlation coefficient was used to assess the correlations between age and the vestibular, cutaneous, and oculomotor thresholds. Statistical significance was set at a 0.05 level.

## Results

Although GVS is mildly unpleasant, no participant reported particular discomfort or withdrew from the study. All participants perceived cutaneous and vestibular sensations and showed oculomotor responses confirmed by VOG recordings (Figure 1).

### Thresholds for cutaneous and vestibular perceptions and oculomotor response

**Figure 2:**
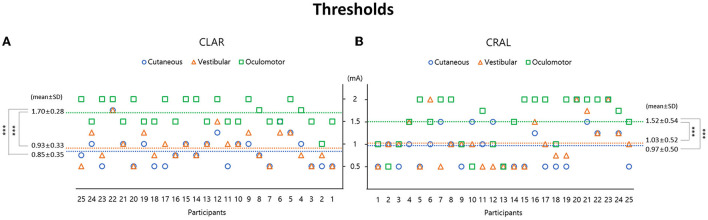
Galvanic vestibular stimulation (GVS) thresholds are presented separately: cutaneous and vestibular perceptions and oculomotor response using two stimulating paradigms of the cathode on the left and anode on the right (CLAR) **(A)** and the cathode on the right and anode on the left (CRAL) **(B)**. In the CLAR paradigm, the oculomotor threshold (1.7 ± 0.28 mA) was significantly higher than the vestibular perception threshold (0.93 ± 0.33 mA, *p* < 0.001, Bonferroni test) and cutaneous perception threshold (0.85 ± 0.35 mA, *p* < 0.001, Bonferroni test) **(A)**. In the CRAL paradigm, the oculomotor threshold (1.52 ± 0.54 mA) was also significantly higher than the vestibular perception threshold (1.03 ± 0.52 mA, *p* < 0.001, Bonferroni test) and cutaneous perception threshold (0.97 ± 0.5 mA, *p* < 0.001, Bonferroni test) **(B)**. GVS threshold values are presented as mean ± standard deviation (mA).

**Figure 3:**
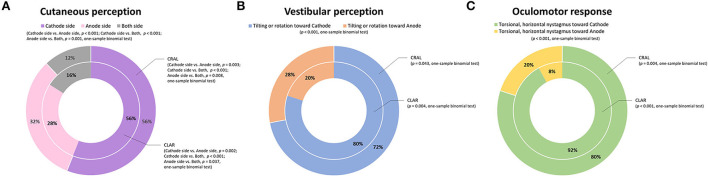
Characteristics of galvanic vestibular stimulation (GVS)-induced cutaneous and vestibular perception and oculomotor responses depending on the stimulating paradigms; the cathode on the left and the anode on the right (CLAR, inside annulus) and the cathode on the right and the anode on the left (CRAL, outside annulus). Cutaneous perceptions were more frequent on the cathode side (56%) than the anode (30%) or both sides (14%; *p* = 0.001, chi-square one-variable test) **(A)**. Rotating and falling sensations were more frequently experienced toward the cathode (76%) than the anode (24%, *p* < 0.001, chi-square one-variable test) direction **(B)**. Nystagmoid ocular movements were observed more frequently with horizontal and torsional nystagmus toward the cathode (86%) than toward the anode (14%, *p* < 0.001, chi-square one-variable test) **(C)**. Values are presented as percentages.

Subjects experienced mild cutaneous tingling or stinging sensations above the mean stimulation intensity of 0.9 ± 0.29 mA (cutaneous threshold, 0.85 ± 0.35 mA on CLAR, and 0.97 ± 0.50 mA on CRAL; Figure 2). These somatosensory perceptions were more frequent on the cathode (56%) than the anode (30%) or both sides (14%; *p* = 0.001, chi-square one-variable test; Figure 3A). Subjects also experienced various vestibular sensations evoked by GVS, as described in the subject's self-report. Some participants experienced dizziness and vertigo, likely the manifestation of whole-body angular rotation or a sense of rotation of the environment as if the objects around them were spinning. Other participants described the feeling of change in gravity. These sensations persisted for several seconds even after cessation of the stimulation above the mean stimulation intensity of 0.98 ± 0.29 mA (vestibular threshold, 0.93 ± 0.33 mA on CLAR, and 1.03 ± 0.52 mA on CRAL; Figure 2). Rotating and falling sensations were more frequently experienced toward the cathode (76%) than the anode (24%, *p* < 0.001, chi-square one-variable test; Figure 3B). The vestibular threshold in males was significantly higher than in females [1 (0.5–1.25) mA vs. 0.75 (0.625–1.125) mA, *Z* = −2.241, *p* = 0.025, Mann–Whitney *U*-test]. However, significant differences were not observed in cutaneous perception and oculomotor response between the sexes. Correlations were not found between age and vestibular, cutaneous, and oculomotor thresholds.

Short-latency eye movements were also observed above the mean stimulation intensity of 1.61 ± 0.35 mA (oculomotor threshold, 1.70 ± 0.28 mA on CLAR, and 1.52 ± 0.54 mA on CRAL; Figure 2). Nystagmoid ocular movements were observed more frequently with horizontal and torsional nystagmus toward the cathode (86%) than toward the anode (14%, *p* < 0.001, chi-square one-variable test; Figure 3C). The mean amplitude and the mean duration of nystagmus were, respectively, 2.03 ± 0.33°/s and 5.01 ± 1.23 s for CRAL and 2 ± 0.43°/s and 5.07 ± 0.98 s for CLAR (Table 3). Notably, the oculomotor threshold [1.75 (1.5–1.75) mA] was significantly higher than the vestibular perception threshold [1 (0.75–1.125) mA, *Z* = −4.866, *p* < 0.001, Mann–Whitney *U*-test] and the cutaneous perception threshold [0.875 (0.75–1.125) mA, *Z* = −5.184, *p* < 0.001, Mann–Whitney *U*-test; χ^2^ = 34.488, *p* < 0.001, Kruskal-Wallis test, Figure 2]. A comparison of the properties of cutaneous and vestibular perception and oculomotor responses between the CRAL and CLAR paradigms revealed no significant differences between them (Table 3).

**Table 3:** Comparison of the properties of cutaneous and vestibular perception and oculomotor response depending on the CRAL and CLAR GVS paradigms.

	**Participants (*****n*** = **25)**		***P*-value**
	**CRAL**	**CLAR**	
**Cutaneous perception**			
Threshold, median (IQR) (mA)	1 (0.5–1.38)	0.75 (0.5–1)	0.542
Intensity, median (IQR) (1–10)	1 (1–2)	1 (1–1.5)	0.176
Nature			1.000
Neutral sensation, *n* (%)	23 (92)	24 (96)	
Painful sensation, *n* (%)	2 (8)	1 (4)	
Site			0.901
Cathode side, *n* (%)	14 (56)	14 (56)	
Anode side, *n* (%)	8 (32)	7 (28)	
Both, *n* (%)	3 (12)	4 (16)	
**Vestibular perception**			
Threshold, median (IQR) (mA)	1 (0.5–1.38)	1 (0.75–1.25)	0.441
Intensity, median (IQR) (1–10)	1 (1–2)	1 (1–2)	0.371
Nature			0.742
Tilting and rotation toward the cathode, *n* (%)	18 (72)	20 (80)	
Tilting and rotation toward the anode, *n* (%)	7 (28)	5 (20)	
**Oculomotor response**			
Threshold, median (IQR) (mA)	1.75 (1–2)	1.5 (1.5–2)	0.075
Degree, median (IQR) (°/s, Rt. eye)	1.9 (1.75–2.35)	2 (1.8–2.3)	0.794
Duration, median (IQR) (s)	4.9 (4.25–5.3)	5 (4.5–5.95)	0.567
Direction			0.417
Horizontal or torsional nystagmus toward the cathode, *n* (%)	20 (80)	23 (92)	
Horizontal or torsional nystagmus toward the anode, *n* (%)	5 (20)	2 (8)	

*CRAL, the cathode on the right and the anode on the left; CLAR, the cathode on the left and the anode on the right; IQR, interquartile range*.

## Discussion

Vestibular and cutaneous perception and oculomotor response thresholds were measured in 25 healthy young subjects during DC GVS with both CRAL and CLAR paradigms, using a two-step approach with gradual increase and decrease in intensities. Vestibular and somatosensory perception thresholds were lower than the oculomotor threshold, indicating the use of the vestibular or somatosensory perception threshold to determine the GVS thresholds in the majority of human trials due to its sensitivity with minimal oculomotor responses. Theoretically, the vestibular threshold might reflect the vestibular function more directly and precisely than other motor response thresholds such as the vestibulo-ocular reflex (VOR) and the vestibulo-spinal reflex (VSR), which may be involved in central adaptation (1, 2, 14, 46, 47). Furthermore, the vestibular threshold could provide a comprehensive assay of peripheral vestibular function including the SSCs or otoliths (48, 49).

A previous study with a similar model reported a vestibular perception threshold of DC of 1.0 ± 0.2 mA, which is very similar to the results of our study (45). Recently, the vestibular threshold was shown to significantly increase after the age of 40 years (49), which has been considered a central gain enhancement for compensating the reduced peripheral vestibular input due to age-related degeneration of vestibular hair cells and afferents (50–52). In particular, prior studies have demonstrated that the aging-related degeneration of vestibular irregular afferent fibers, which are six times more sensitive to GVS than regular fibers, was faster and more potent than those in regular fibers (51, 53). Significant changes, however, have not been observed in the GVS threshold dependent on temporal effects, polarity effects, or body positions (10). Additionally, this study confirmed that there were no significant differences in the specific thresholds between the CRAL and CLAR configurations. Notably, a substantial difference associated with sex was found in vestibular perception but did not occur in cutaneous and oculomotor response thresholds, consistent with a recent trial (54). These sex-related threshold differences were most likely because they have a substantially different size of vestibular apparatus and numbers of vestibular afferent fibers (55, 56). In particular, the total number of myelinated axons in vestibular nuclei is significantly lower in females than in males (55). Regarding the otolith organs, the surface areas of the utricular and saccular maculae, the width of the utricular macula, and the length of the saccular macula are significantly greater in males than in females (56). In addition, diameters of the three SCCs are larger in males than in females, and the difference was statistically significant for the superior SCC (56). These anatomical distinctions might cause vestibular-related behavioral differences. For example, the GVS-induced ocular vestibular-evoked myogenic potential (oVEMP) amplitude was reported higher in males than in females (57). Males were shown to normally perform better in vestibular function-related tasks, in particular, visuospatial cognition such as 3D figures, spatial orientation, and maze navigation (58, 59).

Cutaneous sensations underneath and around the stimulus electrodes, such as burning, tingling, itching, and pain, mostly depend on the stimulus amplitude (14, 33) and have commonly been described as minor adverse effects of GVS intervention. The use of large surface electrodes on the mastoid, covering with electrode gel or topical anesthesia, or controlling the current amplitude helps to minimize the risk of skin irritations, burns, and patient discomfort (14, 60). The cutaneous threshold, also known as the nociceptive threshold, was determined to help manage these disruptions and enhance treatment compliance (17, 26, 61, 62). The cutaneous threshold was determined to be 0.9 mA in this study, with tingling sensation occurring mostly at the cathode side (Figure 3A). The findings were in agreement with a previous study conducted on healthy volunteers utilizing the staircase approach to assess the cutaneous threshold in which a mean value of 0.8 mA was reported (63). Although several previous studies have revealed that a threshold of ~1 mA produced no significant difference in somatosensory sensations between the anode and cathode sides (64–68), the sensory ratings were consistently higher at the cathode than at the anode for pain, tingling, piercing, electrifying, tugging, and pinching senses in other recent studies (Figure 3A) (68, 69). The tingling and itching sensations under the electrodes were usually short lasting and disappeared after a few seconds, similar to our findings. However, in some studies, the sensations were reportedly prolonged (64, 65, 70).

Regarding the characteristics of the vestibular responses to bipolar GVS, rotation and tilt were more frequently experienced toward the cathode (76%) than toward the anode (Figure 3B). Our present findings were in agreement with results from previous studies showing that DC-form GVS induces a tilt of subjective vertical toward the anode and simultaneously induces some illusion of self-tilt or spin toward the cathodal side (8, 71, 72). This dissociation between subjective vertical and body orientation elicited by GVS is mainly determined by central multisensory integration processes involved in the estimation of sensory cue reliability (72). Interestingly, the direction of tilt illusion was associated with posture only when the duration of the GVS was longer than 5 s, and it was toward the anode in freestanding posture, and toward the cathode in an immobilized posture (71). GVS-induced perception of motion is as context-dependent as oculomotor and postural responses, which are specified by the anatomic orientation of the SCC (SCC-driven signals) and the macular surfaces in the otolith organs (otolith-driven signals) (8). Cathodal GVS increases the firing rate of all responsive afferents, and anodal GVS decreases the firing rate of all responsive afferents; consequently, their effects at a canal are represented by a sum of geometric rotational stimuli (9, 40, 73). After calculating both the position of the canals in the plane of the head during upright standing and the incomplete orthogonal of SCCs, the summing vector representing the synergistic effects of SCC afferent activation evoked by GVS from both sides will be a signal roll with a yaw component, both directed toward the cathode, and a perceived visual tilt toward the anode (8, 11, 73). Furthermore, bilateral bipolar GVS produces a utricular firing pattern consistent with a natural stimulus of linear acceleration toward the cathodal side or visual tilt toward the anodal side (8, 11). However, in some psychophysical studies, the otolith contributions to GVS-induced postural responses were negligible compared with the canal input (74, 75).

As GVS can elicit ocular movement in the absence of head movement, it is frequently used to characterize potential pathologies in peripheral and central vestibular signal transmission (14). GVS activates both afferent fibers, including regular and irregular afferents, from all three SCCs to generate a horizontal-torsional nystagmus as a summative effect (14, 76). Regarding the time-related eye movements evoked by the application of a constant current application (DC-GVS) in this study, horizontal nystagmus frequently appeared at the onset of stimuli and immediately subsided, which was consistent with rapid activation but prompt adaptation characterized by thick, irregular fibers. However, non-adapting ocular torsion prevailed for the duration of the stimulus, which was the result of regular SCC fiber activation (14). Due to the oculomotor response threshold as the only method for estimating the GVS threshold in animal trials, it was often used as a control GVS (16, 77). In humans, however, due to discomforts such as oscillopsia and blurred vision caused by ocular movements, measurement of oculomotor threshold is less widely used to trigger vestibular stimulation with GVS. In previous studies with vestibular dysfunction patients, the GVS-induced eye movements were used to evaluate the reduction or absence of oculomotor contribution from the vestibular end organs (60, 78–80). Patients with bilateral vestibular nerve dissection or other pathological disorders affecting both vestibular nerves did not reveal GVS-induced oculomotor responses; therefore, these GVS-induced eye movements are considered a strong predictor of the residual function of the injured vestibular nerve (81–83). In this study, the oculomotor threshold was significantly higher than the vestibular and cutaneous perception thresholds (1.61 mA vs. 0.98 mA and 0.9 mA, Figure 2). Some previous studies have investigated GVS-induced nystagmus in normal subjects (80, 84), in which the oculomotor response appeared with intensities above 1.5 mA, similar to this study (80). Because the entire populations of both SCCs and otolith afferents are susceptible to GVS (76), GVS-induced oculomotor responses are likely to be exceedingly diversified, including the roll, yaw, and pitch components (76). In this study, the most prevalent components were horizontal and torsional nystagmus, primarily beating toward the cathode, compatible with vectored activation of afferent fibers from all three SCCs on one side (51, 76). Other studies have reported obvious GVS-induced oculomotor response of mainly conjugate ocular torsion with the upper pole of both eyes rotating toward the side of the anode, slight skew deviation with hypertropia of the eye ipsilateral to the cathode and hypotropia with the eye of the anode, and small conjugate horizontal eye movements toward the anode (80). Amplitudes of GVS-induced eye movements apparently depend on the stimulating current level or the group of vestibular afferents engaged with regular or irregular firing rates (76, 80, 85). Furthermore, GVS-induced nystagmus was suppressed by visual fixation in light (60, 79, 85), and horizontal nystagmus was mainly observed at the onset and offset of the current step, with ocular torsion prevailing for the entire duration of the stimulus up to several minutes (79).

The DC-GVS is useful for rescuing vestibular function in unilateral vestibular disorders by rebalancing the vestibular deafferentation effect proved in previous studies (77, 86). Therefore, in this study, we investigated only the GVS thresholds using DC. Based on the components of tonic or phasic vestibular fibers that are likely to be recruited for the GVS response, we surmise threshold differences between DC and AC stimulation. Thus, further research is needed to determine the GVS thresholds for the AC (sinusoidal, noisy). Regarding the statistically non-significant results in our study such as age-related GVS threshold, it could be due to a type 2 error related to the small sample size.

In conclusion, GVS is a safe stimulation method when certain standard procedures are followed. Due to the variables influencing GVS-induced cortical excitability, the same quantity of current is likely to have non-uniform effects in each subject with different conditions. Individual factors are a source of heterogeneity in clinical research, which is a significant obstacle for routine use in clinical settings. Therefore, the patient-specific customized threshold should be determined rather than population thresholds. The results of this study show more sensitive vestibular and somatosensory perception thresholds than the oculomotor threshold, which allows the selection of the appropriate current that induces the vestibular effect while simultaneously preventing disturbing ocular movements during the intervention.

## Data availability statement

The original contributions presented in the study are included in the article/supplementary material, further inquiries can be directed to the corresponding author.

## Ethics statement

All experiments were reviewed and approved by the Institutional Review Board at Jeonbuk National University Hospital (No. 2021-07-013-007). The patients/participants provided their written informed consent to participate in this study.

## Author contributions

TTN collected data, conducted the experiment, and wrote the manuscript. J-JK collected data and drew the figures. S-YO designed and supervised the study and wrote the manuscript. All authors contributed to the article and approved the submitted version.

## Funding

This study was supported by a National Research Foundation of Korea (NRF) grant funded by the Korean Government (Ministry of Science and ICT; No. 2022R1A2B5B01001933) and by the Fund of the Biomedical Research Institute, Jeonbuk National University Hospital.

## Conflict of interest

The authors declare that the research was conducted in the absence of any commercial or financial relationships that could be construed as a potential conflict of interest.

## Publisher's note

All claims expressed in this article are solely those of the authors and do not necessarily represent those of their affiliated organizations, or those of the publisher, the editors and the reviewers. Any product that may be evaluated in this article, or claim that may be made by its manufacturer, is not guaranteed or endorsed by the publisher.
